# Prevalence of Mental Distress and Associated Factors among Undergraduate Students of University of Gondar, Northwest Ethiopia: A Cross-Sectional Institutional Based Study

**DOI:** 10.1371/journal.pone.0119464

**Published:** 2015-03-20

**Authors:** Berihun Assefa Dachew, Telake Azale Bisetegn, Resom Berhe Gebremariam

**Affiliations:** 1 University of Gondar, College of Medicine and Health Science, Institute of Public Health Department of Epidemiology and Biostatistics, Gondar, Ethiopia; 2 University of Gondar, College of Medicine and Health Science, Institute of Public Health, Department of Reproductive Health, Gondar, Ethiopia; 3 University of Gondar, College of Medicine and Health Science, Institute of Public Health, Department of Health Education and Behavioral Sciences, Gondar, Ethiopia; Texas Tech University Health Science Centers, UNITED STATES

## Abstract

**Background:**

Mental health problems affect society as a whole and no group is immune to mental disorders; however, students have significantly high level of mental distress than their community peers.

**Objectives:**

The purpose of this study was to assess the prevalence and associated factors of mental distress among undergraduate students of University of Gondar, Northwest Ethiopia.

**Methods:**

Institution based cross sectional study was conducted among 836 students from April 9–11/2014. Stratified multistage sampling technique was used to select the study participants. Data were collected using pretested and structured self-administered questionnaire. Bivariate and multivariate logistic regression model was fitted to identify factors associated with mental distress among students. An adjusted odds ratio with 95% confidence interval was computed to determine the level of significance.

**Results:**

Prevalence of mental distress among students was found to be 40.9%. Female sex (AOR = 1.65; 95% CI 1.17–2.30), lack of interest towards their field of study (AOR = 2.28; 95% CI 1.49–3.50), not having close friends (AOR = 1.48; 95% CI 1.03–2.14), never attend religious programs (AOR = 1.58; 95% CI 1.02–2.46), conflict with friends (AOR = 1.93; 95% CI 1.41–2.65), having financial distress (AOR1.49 = 95% CI 1.05, 2.10), family history of mental illness (AOR = 2.12; 95% CI 1.31–3.45), Ever use of Khat (AOR = 1.71; 95% CI 1.12–2.59), lower grade than anticipated(AOR = 2.07; 95% CI 1.51–2.83), lack of vacation or break (AOR = 1.46; 95% CI 1.06–2.02), and low social support(AOR = 2.58; 95% CI 1.58–4.22) were significantly associated with mental distress.

**Conclusion:**

The overall prevalence of mental distress among students was found to be high. Therefore, it is recommended that mental distress needs due attention and remedial action from policy makers, college officials, non-governmental organizations, parents, students and other concerned bodies.

## Introduction

Mental distress is a mental health problem which includes anxiety, depression, and somatic symptoms such as sleep problems, headache and backache[1–3]

Currently mental distress is an important public health problem and it is a leading cause of disability worldwide, accounting for one third of disability adjusted life years (DALYs) [4, 5]. In Africa mental illness is an important public health challenge that is under recognized as a public burden. Studies conducted in South Africa revealed that the prevalence of common mental disorders is 27% [6] In Ethiopia mental disorders account for 11% of total burden of diseases [7].

Although mental health problems affect society as a whole and no group is immune to mental disorders; students have significantly high level of psychological distress than their community peers [8–10]. This is due to the fact that university students face multiple stressors such as academic load, constant pressure to succeed, competition with peers, financial burden, peer, teacher or parental pressure as well as concerns about the future [11, 12]. This can have negative effects on student’s ability to study and academic outcomes [10, 13, 14]. Such situation of stress may later lead to mental health problems but students seldom seek help for their problems [15–17]. A study among undergraduate students in Canada showed that 30% of students had psychological distresses which was significantly higher than that of adults in the general population of Canada [18]. Furthermore, more than half of students in USA [19] 53% of students in Australia [20] 41.9% of students in Malaysia [21] 10.8% of students in Kenya [22] and 21.6% of students in Ethiopia [23] experienced mental distress. Another study in Ethiopia revealed that 32.6% of medical students experienced mental distress[24]. However, since this study was done only on medical students and with small sample size (273), it may not represent all University students in Ethiopia. Furthermore, this study was done in 2001 which may not reflect the current status of mental distress among University students in Ethiopia.

Even if mental problem was included in the national health policy of Ethiopia, interventions against the problem are very limited and lack of information about the problem is a contributory factor for poor mental health services. Hence, Epidemiological data which shows the burden of mental health problems in students over time is mandatory. Therefore, this study was aimed to determine the prevalence of mental distress and identify the associated factors for mental distress among university students. Results from this study will help in developing evidence based mental health promotion and disease prevention programs.

## Methods

### Study design and period

Institution based cross sectional study was used to determine the prevalence of mental distress and associated factors among University of Gondar undergraduate students from April 9–11/ 2014. The study was conducted in University of Gondar, which is located 748 KM away from Addis Ababa, the capital city of Ethiopia, to the Northwest direction. The study subjects for this study were regular undergraduate students in the University of Gondar. There are about 15,723 regular undergraduate students enrolled in 2013/2014 academic year in the University.

### Sampling technique

Sample size was determined by single population proportion formula using EPI INFO stat calc program with the assumption of population size 15,723, 95% level of confidence, 4% of marginal error, and taking prevalence of mental distress 21.6% [23]. Considering the design effect of two and 10% non response rate, the final sample size became 872. Stratified multistage sampling technique was used to select study participants.

### Data collection tools and procedures

Data were collected using structured self administered questionnaire having five parts. The first part contains socio-demographic characteristics of students. The second part of the questionnaire is a Self Reporting Questionnaire. In this study self reporting questionnaire was used to estimate the prevalence of mental distress in students. This self reporting questionnaire (referred to as the SRQ-20) is a standardized questionnaire having 20 item questions, originally developed by World Health Organization (WHO) designed to indicate mental distress [25]. The tool is adopted from WHO and was validated in low and middle income countries including Ethiopia [26, 27]. In this study, students who were found to have 8 or more symptoms of the 20 items self reporting questionnaires (SRQ-20) in the last 4 weeks were considered as having mental distress. The cutoff point was used based on the reports from the validation study of SRQ-20 that gave the highest sensitivity and specificity which corresponds to a cut-off point of 8 [26]. Third part of the questionnaire is asking about behavioral factors, which includes history of substance use (like Alcohol use, Khat chewing, cigarette smoking, Shisha and sedative use) of students. The fourth part of the questionnaire is assessing about academic factors and the last part of the questionnaire is assessing about social support using 12-item Multidimensional Scale of Perceived Social Support Tool [28]. The items are divided in to factor groups relating to the source of social support namely family, friends and significant others. Each item is scored from one (Very strongly disagree) to 7 (very strongly agree). The total sum of all the 12 items possibly ranges from 12 to 84. A score 69–84 considered as high level of social support whereas a score of 49–68 and 12–48 were considered as moderate and low level of social support respectively. The reliability of the tool was checked using Cronbach’s alpha reliability test with a score of 0.82 (95% CI 0.801–0.837).

One MSc in mental health supervisor and nine nurse professional facilitators were employed and trained for half a day about the purpose of the study, timely collection of data and overall data collection procedure. English version questionnaire was used to collect the data.

### Data quality control

To assure the quality of the data the questionnaire was pre-tested 1 week before the actual data collection time on 50 undergraduate students at Debretabour University and appropriate modification was made. Training was given for the supervisor and the facilitators. During the course of the data collection, facilitators were supervised at each site. The collected data were reviewed and checked for completeness before data entry and incomplete data were discarded.

### Data processing and analysis

Data clean up and cross-checking were done before analysis. Data were checked, coded and entered to EPI INFO version 7 then it was exported to SPSS version 20 for analysis. Both descriptive and analytical statistical procedures were utilized. Descriptive statistics like percentage mean and standard deviation were used for the presentation of demographic data and prevalence of mental distress. Tables were also used for data presentation. Binary logistic regression was used to identify factors associated with mental distress among the students. Multiple logistic regression model was fitted to control the possible effect of confounders and finally the variables which had independent association with mental distress were identified on the basis of OR, with 95%CI and p-value less than 0.05. The variables were entered to the multivariate model using the Backward Stepwise regression method. Model fitness was checked using Hosmer and Lemeshow goodness of a fit test (P = 0.77).

### Ethics statement

The study was approved by the Institute Review Board **(IRB)** of the University of Gondar. Written Informed consent was obtained from respondents who were selected to participate in the study.

## Results

### Socio demographic characteristics of the respondents

Out of 872 study participants, 836 students participated in the study giving response rate of 95.87%.

Most 538 (64.4%) respondents were male. The mean (standard deviation) age of the respondents was 20 (±1.54) years. The higher percentages of the respondents were from urban background 544 (65.1%). About three fourth of the respondents 618 (73.9%) were followers of Orthodox Christianity and majority of the respondents 714 (85.5%) take part in religious practice. About 270 (32.3%) of the respondents were from faculty of technology and more than one third of the respondents 286 (34.2%) were first year in academic enrollment. Out of the total respondents 675(80.7%) joined the department by their choice and most 706 (84.4%) were currently interested in their felid of study. Five hundred twenty one (62.3%) of the respondents had pocket money ranging from minimum of 50 to maximum of 2000 Ethiopian birr per month and 97(11.6%) of the respondents had family history of mental illness (Table 1).

**Table 1:** Socio demographic characteristics of the respondents, University of Gondar, Northwest Ethiopia, April 2014 (n = 836).

Variables		Frequency	Percent (%)
Age in years	≤19	160	19.1
	20–24	658	78.7
	25+	18	2.2
Sex	Male	538	64.4
	Female	298	35.6
Residence	Urban	544	65.1
	Rural	292	34.9
Religion	Orthodox Christian	618	73.9
	Protestant	124	14.8
	Muslim	78	9.3
	Catholic	11	1.3
	Other*	5	0.6
College/faculty/school	Technology	270	32.3
	Business and economics	130	15.5
	Medicine and health sciences	101	12.1
	Natural computational sciences	100	12.0
	Social sciences and humanities	77	9.2
	Law	76	9.1
	Agriculture	30	3.6
	Education	30	3.6
	Veterinary medicine	22	2.6
Department choice	Preferred	675	80.7
	Not preferred	161	19.3
Year of enrollment	1^st^	286	34.2
	2^nd^	250	29.9
	3^rd^	256	30.6
	4^th^ and above	44	5.3
Have pocket money	Yes	521	62.3
	No	315	37.7
Family history of mental illness	Yes	97	11.6
	No	739	88.4

Other* stands for: 2 Jubbah witness, 1 waqefata and 2 no religion

### Prevalence of mental distress

Prevalence of mental distress among students was found to be 40.9% (95% CI 37.6, 44.1%). Relatively high (44.6%) prevalence of mental distress was found among female students as compared to males (38.8%). The distribution of SRQ-20 showed a mean score of 6.53 (±4.14) ranging from 0 to 20.

### Factors associated with mental distress

In multivariate logistic regression analysis, sex, interest towards the department, having close friends, religious practice, conflict with friends, absence of pocket money, having financial distress, family history of mental illness, ever use of Khat, decrease grade than anticipated, lack of vacation or break, and social support were significantly associated factors of mental distress among respondents (Table 2).

**Table 2:** Bivariate and multivariate logistic regression analysis of factors associated with mental distress among students, University of Gondar, Northwest Ethiopia, April 2014 (n = 836).

Variables		Mental distress		OR with 95% CI	
		Yes	No	Crude	Adjusted
Sex	Male	209	329	1	1
	Female	133	165	1.26 (0.95–1.69)	1.65 (1.17, 2.30)*
Department choice	Preferred	264	411	1	1
	Not preferred	78	83	1.46 (1.04, 2.07)	1.13 (0.70, 1.83)
Interest to department	Yes	268	438	1	1
	No	74	56	2.16 (1.48, 3.15)	2.28(1.49, 3.50) *
Having close friends	Yes	248	410	1	1
	No	94	84	1.85 (1.33, 2.56)	1.48 (1.03, 2.14) *
Religious practice	Yes	281	433	1	1
	No	61	61	1.54 (1.05, 2.26)	1.58 (1.02, 2.46) *
Ever had conflict	Yes	226	241	2.04 (1.54, 2.72)	1.93 (1.41, 2.65)*
	No	116	253	1	1
Pocket money	Yes	189	332	1	1
	No	153	162	1.65 (1.24, 2.21)	1.53 (1.09, 2.14) *
Financial distress	Yes	154	142	2.03 (1.52, 2.71)	1.49(1.05, 2.10) *
	No	188	352	1	1
Family history of mental illness	Yes	60	37	2.62 (1.70, 4.06)	2.12 (1.31, 3.45) *
	No	282	457	1	1
Ever use of Khat	Yes	81	69	1.91 (1.34, 2.73)	1.71(1.12, 2.59) *
	No	261	425	1	1
Current use of Khat	Yes	63	51	1.96 (1.32, 2.92)	1.13 (0.59, 2.19)
	No	279	443	1	1
Ever use shisha	Yes	25	18	2.08 (1.12, 3.88)	2.01(0.96, 4.21)
	No	317	476	1	1
Current use of shisha	Yes	22	17	1.92 (1.23, 4.32)	1.33 (0.47,3.70)
	No	320	477	1	1
Decrease grade than anticipated	Yes	205	194	2.31 (1.71, 3.06)	2.07(1.51,2.83) *
	No	137	300	1	1
Missed many class	Yes	54	44	1.92 (1.25, 2.93)	1.46 (0.91, 2.38)
	No	288	450	1	1
Anticipation of graduation	Yes	186	221	1.47 (1.12, 1.94)	1.21 (0.87,1.67)
	No	156	273	1	1
Lack of vacation	Yes	156	161	1.74 (1.31, 2.31)	1.46(1.06, 2.02) *
	No	186	333	1	1
Social support	Low	152	170	3.10 (1.97, 4.86)	2.58(1.58, 4.22) *
	Moderate	158	212	2.58 (1.65, 4.03)	2.50(1.54, 4.04) *
	High	32	111	1	1

* = Statistically significant at P<0.05

## Discussion

The prevalence of mental distress among students was found to be 40.9% in the current study. This finding is lower as compared to studies in USA (57%) [19] Australia (53%) [20] and Brazil (44.7%) [29]. The difference could be attributed to the socio, cultural and environmental factors. It was also lower when compared with study in Hawassa University, Ethiopia 49.1% [30]. This might be due to time variation; the improvement of infrastructure and a service option provided by school authorities from time to time.

However, the prevalence in the current study was higher when compared with studies in France (25.7%) [31], Norway (22.9%) [32], Iceland (22.5%) [33] and Australia (19.2%) [10]. This could be due to the different instrument used in other studies or it could be a real difference. However, nearly similar prevalence was reported in Malaysia’s study (41.9%) [21].

The prevalence of mental distress in this study was higher among female students as compared to their male counterparts. Similarly; the likelihood of mental distress was higher among female students as compared to males (AOR = 1.65; 95% CI 1.17–2.30). The finding is consistent with other studies in Australia [9, 10], France [13] Norway [32] and Turkey [34]. The affective nature of their response to stressors, domestic violence, and hormonal changes during menstruation could be the possible causes for the higher prevalence of mental distress among female students [35].

Although, the current study does not show any significant association between year of study and mental distress, other studies done in Australia [8], Saudi Arabia [36] Norway [31] and Ethiopia (Hawassa) [37] identified that freshman (first year) students were more likely to have mental distress than second year and above. This may be due to the fact that, first year students face difficulty in adapting to University education.

In this particular study; having interest towards the field of study was an important factor of mental distress. Students who were not interested in their field of study were two times more likely to experience mental distress as compared with those who were interested with their department (AOR = 2.28; 95% CI 1.49–3.50). A study among students in Adama University also came up with the same finding [23]. Moreover, The odds of mental distress by respondents who had family history of mental illness were 2.12 times higher as compared with those who have not (AOR = 2.12; 95% CI 1.31–3.45) the finding is in line with study in Adama, Ethiopia [23]. This could be explained by genetic predisposition and living conditions within the families. In addition, caring for the mentally ill family member may also be an additional stress that contributes to a higher prevalence of mental distress [4]. Moreover, the study revealed that financial distress was strongly associated with mental distress. Those students who have financial distress and have no pocket money were more likely to experience mental distress (AOR = 1.53; 95% CI 1.09–2.14). This finding is supported by other studies in Australia [8], United States of America [38]and Nigeria [39] which found that financial hardship was independently associated with mental distress. The rising cost of stationary materials and photocopy services may crate stressful situation in students. Moreover, students with financial difficulty experience anxiety, frustration, and sense of haplessness and trouble of sleeping which may further lead students mentally distressed [40]. However, in this study; there was no significant association between low amount pocket money and mental distress in students.

The study also found that mental distress was significantly associated with religious practice. Students who were involved in religious program, irrespective of their religion, were less likely to be mentally distressed. This finding is supported by the other studies done in Ethiopia [23, 37]. This could be due to the fact that religious teaching helps in stress management. Furthermore, it facilitates the development of adaptive behaviors [41, 42]

On the other hand, students who ever had conflict with their room mates were mentally distressed (AOR = 1.93; 95% CI 1.41–2.65). This finding is consistent with a study in Adama University [23]. This might be due to the fact that, campus life where students live together in a group; conflicts may break social ties and might result in a stressful situation.

In addition, ever use of khat was found to be a significant factor of mental distress. Students who ever use khat were 1.7 times more likely to have mental distress as compared to students who never use khat. This finding is in line with other studies in Ethiopia [23, 43]and Sao Paulo, Southeastern Brazil [44]. This may be due to the fact that substance use leads to inefficiency in life function, impaired relationship and sleep difficulty. Furthermore, substance use is associated with increased absenteeism from class and poor cadmic performance which can further lead to mental distress in students [45]. However, since the study is a cross-sectional, it is difficult to ascertain the direction of causality.

Academic factors were found to be other factors associated with mental distress. Students whose grade was lower than anticipated were two times more likely to have mental distress than their counter parts (AOR = 2.07; 95% CI 1.51–2.83). However, the present study did not show any association between mental distress and academic performance.

In this study, social support was also found to be another determinant factor for mental distress in students. Having high level of social support from significant others were negatively associated with mental distress. In this study, students with low social support were more than two times more likely to have mental distress as compared to those students with high social support AOR = 2.58; 95% CI 1.58–4.22). This finding is also supported by other studies [32, 44, 46]. This could be due to its effect on hypothalamic pitutary adrenocortical (HPA) system in reducing genetic and environmental vulnerabilities. Furthermore, It is also important for maintaining good physical and mental health [47].

### Limitation of the study

This study has some important limitations that should be kept in mind when interpreting the results. First, the cross-sectional nature of the study design does not confirm definitive cause and effect relationship. Furthermore, the study may prone to reporting bias since the data was collected based on self-reported information. Finally; reports for some of the questions were past history or encounters which are prone to recall bias.

## Conclusion and Recommendation

The prevalence of mental distress among students was found to be high. The prevalence of mental distress was relatively high among female students. Being female sex, lack of interest towards the field of study, not having close friends, never attend religious programs, conflict with friends, absence of pocket money, having financial distress, family history of mental illness, ever use of Khat, lower grade than anticipated, lack of vacation or break, and low to moderate social support were factors associated with mental distress. Therefore, it is recommended that mental distress needs due attention and remedial action from policy makers, college officials, non-governmental organizations, parents, students and other concerned bodies. Programs aimed at preventing mental distress need to address these identified factors of mental distress against students.

## References

[1] Giang KB, Dzung TV, Kullgren G, Allebeck P. Prevalence of mental distress and use of health services in a rural district in Vietnam. Glob Health Action 2010; 15:3. 10.3402/gha.v3i0.2025 PMC2837473 20228898

[2] Rocha SV, de Almeida MG, de Araújo TM, Júnior JSV. Prevalence of common mental disorders among the residents of urban areas in Feira de Santana, Bahia. Rev Bras Epidemiol 2010; 13(4):1–11. 10.1590/s1415-790x2010000400008 21180852

[3] Gelder Michael A N, Lopez-Ibor Juan, Geddes John. Oxford Textbook of Psychiatry (2nd edn), SIMS. A. Symptoms in the Mind. An Introduction to Descriptive Psychopathology. London: BailliÃ¨re-Tindall: Oxford University Press.

[4] Begg S, Vos T, Barker B, Stevenson C, Stanley L, &, Lopez AD. The burden of disease and injury in Australia. Canberra: AIHW; 2007.

[5] World Health Organization. The global burden of disease: In. Geneva; 2008.

[6] Havenaar JM, Geerlings MI, Vivian L, Collinson M, Robertson B. Common mental health problems in historically disadvantaged urban and rural communities in South Africa: prevalence and risk factors. Soc Psychiatry Psychiatr Epidemiol 2007; 7 (1007):294–299. 10.1007/s00127-007-0294-9 18058040

[7] Abdulahi H, Haile Mariam D, Kebede D. Burden of disease analysis in rural Ethiopia. Ethiopian Medical journal 2001; 39(4):271–281. 12380227

[8] Cvetkovski S, Reavley NJ, Jorm AF. The prevalence and correlates of psychological distress in Australian tertiary students compared to their community peers. Aust N Z J Psychiatry 2012; 46(5):457–467. 10.1177/0004867411435290 22535294

[9] Leahy CM, Peterson RF, Wilson IG. Distress levels and self-reported treatment rates for medicine, law, psychology and mechanical engineering students: cross-sectional study. Australian and New Zealand Journal of Psychiatry 2010; 44:608–615. 10.3109/00048671003649052 20560848

[10] Stallman HM. Psychological distress in university students: a comparison with general population data. Australian Psychologist 2010; 45(45):249–257.

[11] Sreeramareddy CT, Shankar OR, Binu VS, Mujhopadhyay C, Ray B, Menezes RG. Psychological morbidity, sources of stress and coping strategies among undergraduate medical students in Nepal. BMC Med Educ 2007; 7:26 17678553 10.1186/1472-6920-7-26 PMC1951961

[12] Vaez M, Ponce de Leon A, Laflamme M. Health related determinants of perceived quality of life: a comparison between first year university students and their working peers. 2006; 26:167–177. 16477109

[13] Verger P, Gilbert F, Kovess-Masfety V. Psychiatric disorders in students in six French universities: 12-month prevalence, comorbidity, impairment and help-seeking. Social Psychiatry and Psychiatric Epidemiology 2010;45:189–199. 10.1007/s00127-009-0055-z 19381424

[14] Dahlin M, Joneborg N, Runeson B. Stress and depression among medical students: a cross-sectional study. Med Educ 2008; 39:594–604. 10.1111/j.1365-2929.2005.02176.x 15910436

[15] Alzahem AM, Van der Molen HT, Alaujan AH, Schmidt HG, Zamakhshary MH. Stress amongst dental students: a systematic review. Eur J Dental Educ 2011; 15 8–18. 10.1111/j.1600-0579.2010.00640.x 21226800

[16] Galbraith ND, Brown K. Assesing intervention effectiveness for reducing stress in student nurses: quantitative systemic review. J Advanced Nursing 2011; 67:709–721. 10.1111/j.1365-2648.2010.05549.x 21214619

[17] Tyseen R, Vaglum P, Gronvold NT, Ekeberg O. Factors in medical school that predict postgraduate mental health problems in need of treatment. A nationwide and longitudinal study. Med Educ 2010; 35:110–120. 10.1046/j.1365-2923.2001.00770.x 11169082

[18] Adlaf E.M, Gliksman L, Demers A. The prevalence of elevated psychological distress among Canadian undergraduates. Journal of American College Health 2007; 50 67–72. 10.1080/07448480109596009 11590985

[19] Mosley TH, Perrin SG, Neral SM, Dubbert PM. Stress, coping, and well-being among third-year medical students. Acad Med, 2008;69(9):765–767. 10.1097/00001888-199409000-00024 8074778

[20] Stallman HM. Prevalence of psychological distress in university students—implications for service delivery Aust Fam Physician 2008; 37(8):673–677. 18704221

[21] Mohd Sidik Sherina, Rampal Lekhraj, Kaneson N. Prevalence of emotional disorders among medical students in a Malaysian university. Asia Pacific Family Medicine 2008; 2(4):213–217.

[22] Jenkins R, Njenga F, Okonji M, Kigamwa P, Baraza M, Ayuyo J, et al Prevalence of Common Mental Disorders in a Rural District of Kenya, and Socio-Demographic Risk Factors *International Journal of Environmental Research and Public Health* 2012; 9:1810–1819. 10.3390/ijerph9051810 22754474 PMC3386589

[23] Dessie Y, Ebrahim J, Awoke T. Mental distress among university students in Ethiopia: a cross sectional survey. Pan Afr Med J 2013; 15:95 10.11604/pamj.2013.15.95.2173 24198889 PMC3810159

[24] Alem A, Araya M, Melaku Z, Wendimagegn D, Abdulahi A. Mental distress in medical students of Addis Ababa University. Ethiopian medical journal 2005; 43(3):159–166. 16370547

[25] World Health Organization: A users guide to the Self Reporting Questionnaire (SRQ) Geneva;WHO 1994.

[26] Youngmann R, Zilber N, Workneh F, Giel R. Adapting the SRQ for Ethiopian populations: a culturally-sensitive psychiatric screening instrument. Transcult Psychiatry 2008; 45(4):566–589. 10.1177/1363461508100783 19091726

[27] Lund C, Silva MD, Plagerson S, Cooper S, Chisholm D, Das J, et al Poverty and mental disorders: breaking the cycle in low- income and middle- income countries. Lancet 2011; 378 1502–1514. 10.1016/S0140-6736(11)60754-X 22008425

[28] Zimet GD, Powell SS, Farley GK, Werkman S and Berkoff KA. Psychometric characterstis of the Multidimentional Scale pf Percived Sociak Support *journal of Personality Assessment* 1990; 55:610–617. 2280326 10.1080/00223891.1990.9674095

[29] Maria CP, Lima SD, Ana TD, Abreu RC. Prevalence and risk factors of common mental disorders among medical students. Rev Saúde Pública 2006; 40(6):1035–1041. 17173160 10.1590/s0034-89102006000700011

[30] Tesfaye A. Prevalence and correlates of mental distress among regular undergraduate students of Hawassa University: a cross sectional survey. East Afr J Public Health 2009; 6(1):85–94. 20000071 10.4314/eajph.v6i1.45755

[31] Verger P, Combes JB, Kovess-Masfety V. Psychological distress in first year university students: socioeconomic and academic stressors, mastery and social support in young men and women. Social Psychiatry and Psychiatric Epidemiology 2009; 44:643–650. 10.1007/s00127-008-0486-y 19096741

[32] Nerdrum P, Rustøen T, Rønnestad M. Student psychological distress: a psychometric study of 1750 Norwegian 1st-year undergraduate students. Scandinavian Journal of Educational Research 2006; 50: 95–109.

[33] Bernhardsdottir J, Vilhjalmsson R. Psychological distress among university female students and their need for mental health services. J Psychiatr Ment Health Nurs 2013; 20(8):672–678. 10.1111/jpm.12002 22988953

[34] Bayram N, Bilgel N.The prevalence and socio-demographic correlations of depression, anxiety and stress among a group of university students. Social Psychiatry and Psychiatric Epidemiology 2008; 43: 667–672. 10.1007/s00127-008-0345-x 18398558

[35] Pagel MD, Erdly WW, Becker J. Social networks: We get by with (and in spite of) a little help from our friends *Journal of Personality and Social Psychology* 1987; 53 793–804. 3681652 10.1037//0022-3514.53.4.793

[36] Abdulghani HM, AlKanhal AA, Mahmoud ES, Ponnamperuma GG, Alfaris EA. Stress and its effects on medical students: a cross-sectional study at a college of medicine in Saudi Arabia. Journal of health, population, and nutrition 2011; 29(5):516–522. 22106758 10.3329/jhpn.v29i5.8906 PMC3225114

[37] Ovuga Emilio, Boardman Jed, Wasserman Danuta. Undergraduate student mental health at Makerere University, Uganda. World Psychiatry 2006; 5:51–52. 16757997 PMC1472270

[38] Eisenberg D, Gollust SE, Golberstein E. Prevalence and correlates of depression, anxiety, and suicidality among university students. American Journal of Orthopsychiatry 2007; 77:534–542. 10.1037/0002-9432.77.4.534 18194033

[39] Omigbodun OO, Odukogbe AT, Omigbodun AO, Yusuf OB, Bella TT, Olayemi O. Stressors and psychological symptoms in students of medicine and allied health professions in Nigeria. Soc Psychiatry Psychiatr Epidemiol 2006; 41(5):415–421. 16479325 10.1007/s00127-006-0037-3

[40] Braxton JM., Bray NJ, Sullivan AS. The Influences of Stress-Related Coping Strategies of College Student Departure Decisions. Journal of College Student Development 1999; 40(6):645–657.

[41] Nabi Naheed, Yousuf Aisha, Iqbal Azam. Prevalence of anxiety and depression among doctors working in a Private Hospital in Pakistan. ASEAN Journal of Psychiatry 2012; 13:1.

[42] Goebert D, Thompson D, Takeshita J, Beach C: Depressive symptoms in medical students and residents: a multischool study. Acad Med 2009; 84(2):236–241. 10.1097/ACM.0b013e31819391bb 19174678

[43] Damena T, Mossie A, Tesfaye M. Khat chewing and mental distress: a community based study, in jimma city, southwestern ethiopia. Ethiop J Health Sci 2011; 21(1):37–45. 22434984 10.4314/ejhs.v21i1.69042 PMC3275849

[44] Gallagher RP. National survey of counseling center directors. Monograph The International Association of Counseling Services. 2010.

[45] Ningombam S, Hutin Y, Murhekar MV. Prevalence and pattern of substance use among the higher secondary school students of Imphal, Manipur, India. Natl Med J India 2011; 24:11–15. 21608351

[46] Storrie Kim, Ahern Kathy, Tuckett Anthony. A systematic review: Students with mental health problems: A growing problem. International Journal of Nursing Practice 2010; 16(1):1–6. 20158541 10.1111/j.1440-172X.2009.01813.x

[47] Ozbay F, Johnson DC, Dimoulas E, Morgan CA, Charney D, Southwick S. Social support and resilience to stress: from neurobiology to clinical practice. Psychiatry (Edgmont (Pa: Township)) 2007; 4(5):35–40. 20806028 PMC2921311

